# Constructing an integrated gene similarity network for the identification of disease genes

**DOI:** 10.1186/s13326-017-0141-1

**Published:** 2017-09-20

**Authors:** Zhen Tian, Maozu Guo, Chunyu Wang, LinLin Xing, Lei Wang, Yin Zhang

**Affiliations:** 1 School of Computer Science and Engineering, Harbin Institute of Technology, Harbin, 150001 People’s Republic of China; 2 Institute of Health Service and Medical Information Academy of Military Medical Sciences Beijing, Beijing, 100850 China

**Keywords:** Gene Ontology, Gene similarity networks, Similarity network fusion, Disease gene identification

## Abstract

**Background:**

Discovering novel genes that are involved human diseases is a challenging task in biomedical research. In recent years, several computational approaches have been proposed to prioritize candidate disease genes. Most of these methods are mainly based on protein-protein interaction (PPI) networks. However, since these PPI networks contain false positives and only cover less half of known human genes, their reliability and coverage are very low. Therefore, it is highly necessary to fuse multiple genomic data to construct a credible gene similarity network and then infer disease genes on the whole genomic scale.

**Results:**

We proposed a novel method, named RWRB, to infer causal genes of interested diseases. First, we construct five individual gene (protein) similarity networks based on multiple genomic data of human genes. Then, an integrated gene similarity network (IGSN) is reconstructed based on similarity network fusion (SNF) method. Finally, we employee the random walk with restart algorithm on the phenotype-gene bilayer network, which combines phenotype similarity network, IGSN as well as phenotype-gene association network, to prioritize candidate disease genes. We investigate the effectiveness of RWRB through leave-one-out cross-validation methods in inferring phenotype-gene relationships. Results show that RWRB is more accurate than state-of-the-art methods on most evaluation metrics. Further analysis shows that the success of RWRB is benefited from IGSN which has a wider coverage and higher reliability comparing with current PPI networks. Moreover, we conduct a comprehensive case study for Alzheimer’s disease and predict some novel disease genes that supported by literature.

**Conclusions:**

RWRB is an effective and reliable algorithm in prioritizing candidate disease genes on the genomic scale. Software and supplementary information are available at http://nclab.hit.edu.cn/~tianzhen/RWRB/.

## Background

Prioritization of candidate disease genes is a fundamental challenge in human health with applications to understand disease mechanisms, diagnosis and therapy [1–5]. Many human diseases are complex and polygenic, involving linking genomic variation to clinical phenotype. Traditional linkage analyses and association study have conducted susceptible genomic interval in the chromosomes [6–8]. However, since the susceptible locus may contain several hundreds of genes, computational approaches are widely accepted to further infer causal genes that are associated with interested diseases [9–11].

Given a disease and its disease genes, the target of prioritization is usually to measure the similarity between candidate genes and the disease genes [1, 12, 13]. It is generally believed that it is the abnormal expression of disease genes that lead to the diseases happen. The disease genes are also called causal genes or disease related genes for the diseases sometimes. Many methods which take the “guilt by association” principle have been proposed to prioritize candidate genes based on a comprehensive range of biological information [10, 14–21]. They are devoted to fully characterize genes (or corresponding gene products), to measure the similarity between known disease genes and candidate genes more precisely and reliably. These methods are usually called feature-based methods [22]. The metric of similarity is generally based on sequence-based features of genes [23–25], functional annotation of genes [13, 26, 27] and protein-protein interaction data [28, 29]. The ultimate goal is to discriminate disease genes and non-disease genes based on certain characteristics of genes [30, 31].

More recently, many methods [32–38] make use of phenotype similarity between diseases to prioritize candidate disease genes [39, 40]. This is because phenotypic similarity of diseases can help increase the total number of known disease genes for less studied disease phenotypes [41]. The underlying assumption for these methods is that similar phenotypes are caused by functionally related genes [12, 42]. These methods are usually called similarity-based methods [22]. Lage [2] built a Bayesian model based on PPI network and phenotype similarity network, and then prioritized the candidate genes with the help of candidate protein complex. Kohler [32] first grouped diseases into families and then employed a random walk from known disease genes in its family to prioritize candidate genes. Later, Wu [33] put forward a regression model, named CIPHER, to exploit phenotype-gene associations. More recently, Li [35] first constructed a heterogeneous network by making the best use of the phenotype similarity network and gene network as well as the phenotype-gene relationship information. Then they employed the random walk model, called RWRH, to infer disease genes.

Most methods for prioritizing candidate disease genes above mainly rely on PPI networks. However, current PPI networks mainly have two shortcomings. One is that the coverage of the available PPI networks is typically low [29, 43, 44]. Since the curated physical interactions are generally preferred, they often lead to insufficient coverage in human genome [45]. This may result in a serious problem that some known disease genes cannot be mapped into the PPI networks. To address this issue, several researchers [6, 46–48] have attempted to construct gene semantic similarity network. For instance, Li [6] employ a random walk with restart algorithm on the multigraphs, which merges various genomic networks to enlarge the range of candidate genes and increase the noise tolerance of networks. However, these different genomic networks do not integrate indeed. The weights assigned to different networks are also difficult to confirm.

The other is the low reliability of PPI networks [49]. Since a single data source is prone of bias and incompleteness, integration of various genomic data sources is highly demanded for the study of disease gene prioritization [6, 10, 50, 51]. Although multiple data sources are available, most methods only access one or two of these databases, which all have their limitations. Chen [52] proposed a method, called BRIDGE, which utilize a multiple regression model with lasso penalty to prioritize the candidate genes by integrating disease phenotype similarity. Zhang [53] adopted a Bayesian regression approach to integrate multiple PPI networks. The approach takes the strength of association between a query disease and a candidate gene as a score to prioritize candidate genes. However, to the best of our knowledge, constructing and integrating multiple gene similarity networks for prioritizing disease genes has not been investigated well. As a result, there is still a need for the improvement in these disease gene prioritization methods.

**Fig. 1:**
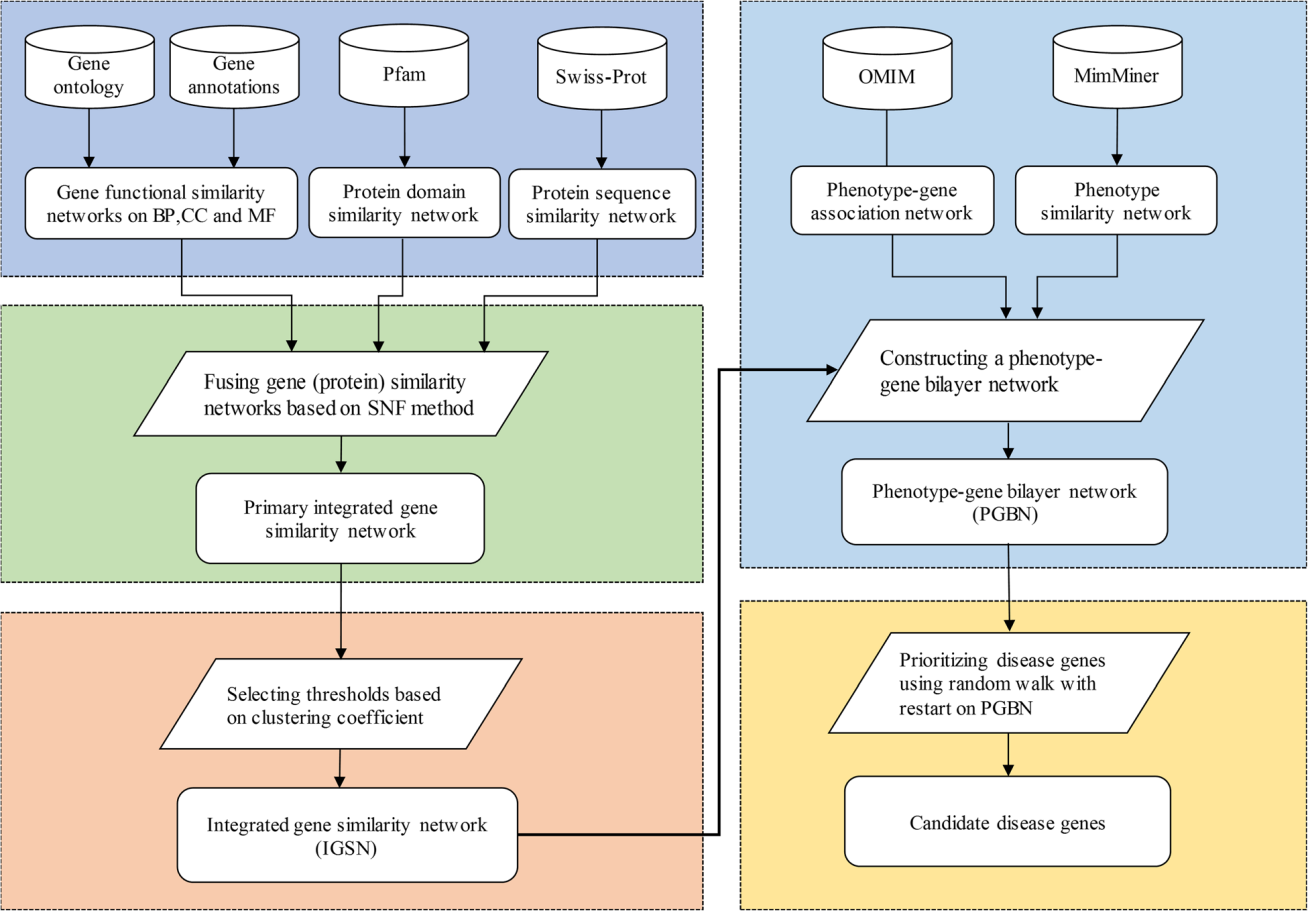
The flow chart of the proposed method

Motivated by the observations above, we proposed the **random walk with restart on phenotype-gene bilayer network** (RWRB) algorithm to prioritize candidate genes of diseases. We firstly construct five individual gene similarity networks based on genomic data of genes. Then we obtain an **integrated gene similarity network** (IGSN) via the **similarity network fusion** (SNF) method. After that, combining the phenotype similarity network, phenotype-gene association network and IGSN, a phenotype-gene bilayer network is constructed. In the end, we employ the RWRB algorithm on the phenotype-gene bilayer network and prioritize candidate disease genes on the whole genomic scale. On the benchmark datasets, RWRB performs better than other leading approaches. The framework of our proposed method is shown in Fig. 1. It is noteworthy that, to take advantage of more abundant genome data related to genes, we treat sequence and domain similarity between proteins as the similarity between their corresponding protein-coding genes. Therefore, the similarity between genes or proteins is collectively called gene similarity to simplify in this article.

## Methods

### Datasets

#### Phenotype similarity network

In OMIM database, a phenotype is defined as a MIM record. The similarity between phenotypes has been calculated by text mining of MIM records [54]. We downloaded the phenotype similarity network [39], which contains pairwise similarity scores for 5080 phenotypes, covering the majority of recorded human phenotypes in this database.

#### Phenotype-gene association network

The phenotype-gene relationship data is downloaded from the OMIM database (http://omim.org/). After filter out phenotypes which do not belong to the phenotype similarity network above and have no known disease genes, we collect 2133 phenotypes and 1893 disease genes involving 2386 phenotype-gene associations totally.

#### Gene data

Gene Ontology (GO) and Gene Ontology Annotation (GOA) data of human is download from the GO website (http://geneontology.org, dated November 2, 2015). The numbers of annotated genes in cellular component (CC), molecular function (MF) and biological process (BP) ontologies are 16,938, 18,225, and 17,072, respectively. Here, we consider all types of annotations which contains Inferred from Electronic Annotations. Amino acid sequences of proteins are obtained from the UniProt database [55]. The number of protein sequence in human database is 18,830. Domains of proteins are downloaded from PFAM database (http://www.sanger.ac.uk/Software/Pfam) [56]. Here, we only collected Pfam-A, a collection of manually curated and functionally assigned domains, instead of Pfam-B, which is computationally derived collection of domains, to ensure accuracy in measuring the similarity between proteins. The number of human proteins annotated by Pfam-A is 18,523 involving 5333 kinds of domains in this database.

### Construction of gene similarity networks based on genomic data of genes

#### Constructing gene functional similarity networks based on gene ontology

GO is a standardized and controlled vocabulary to describe genes and gene product attributes. It comprises three orthogonal ontologies: CC, MF and BP, respectively. In our research, CC, MF and BP ontology has 3817, 9943 and 27,864 terms, respectively.

Functional similarity between genes can be inferred from the semantic relationships of their GO terms [51, 57]. In this work, the functional similarity between two genes is measured by Wang method [58] taking BMA strategy because of its an outstanding performance. For the sake of three ontologies are independent, the functional similarity between genes can be measured from three different ontologies. Therefore, we obtain network on CC, MF and BP ontology, respectively.

#### Constructing protein similarity network based on protein sequence

We used bitscores calculated by the Basic Local Alignment Search Tool (BLAST) to create our sequence homology dataset. First of all, we performed an all-versus-all comparison between proteins with an expectation-value threshold of 10^−6^. Then, the similarity between proteins was normalized according to their corresponding bitscores of proteins. Then, applying this operation to all protein pairs, we got the similarity network of protein sequences.

#### Constructing protein similarity network based on protein domains

We calculated the Jaccard scores [59] between protein domain set as domain similarity of proteins. The Jaccard score between proteins *p*_1_ and *p*_2_ is defined as ${D}_{p_1}\cap {D}_{p_2}/{D}_{p_1}\cup {D}_{p_2}$, which is the ratio of the number of common domains between *p*_1_ and *p*_2_ over the total number of domains in *p*_1_ and *p*_2_. *D*_*p*_ denotes the domain set of protein*p*. There are totally 18,526 proteins involving 5333 kinds of domain used in our analysis. Applying this operation to all protein pairs, thus we constructed a domain similarity network.

**Fig. 2:**
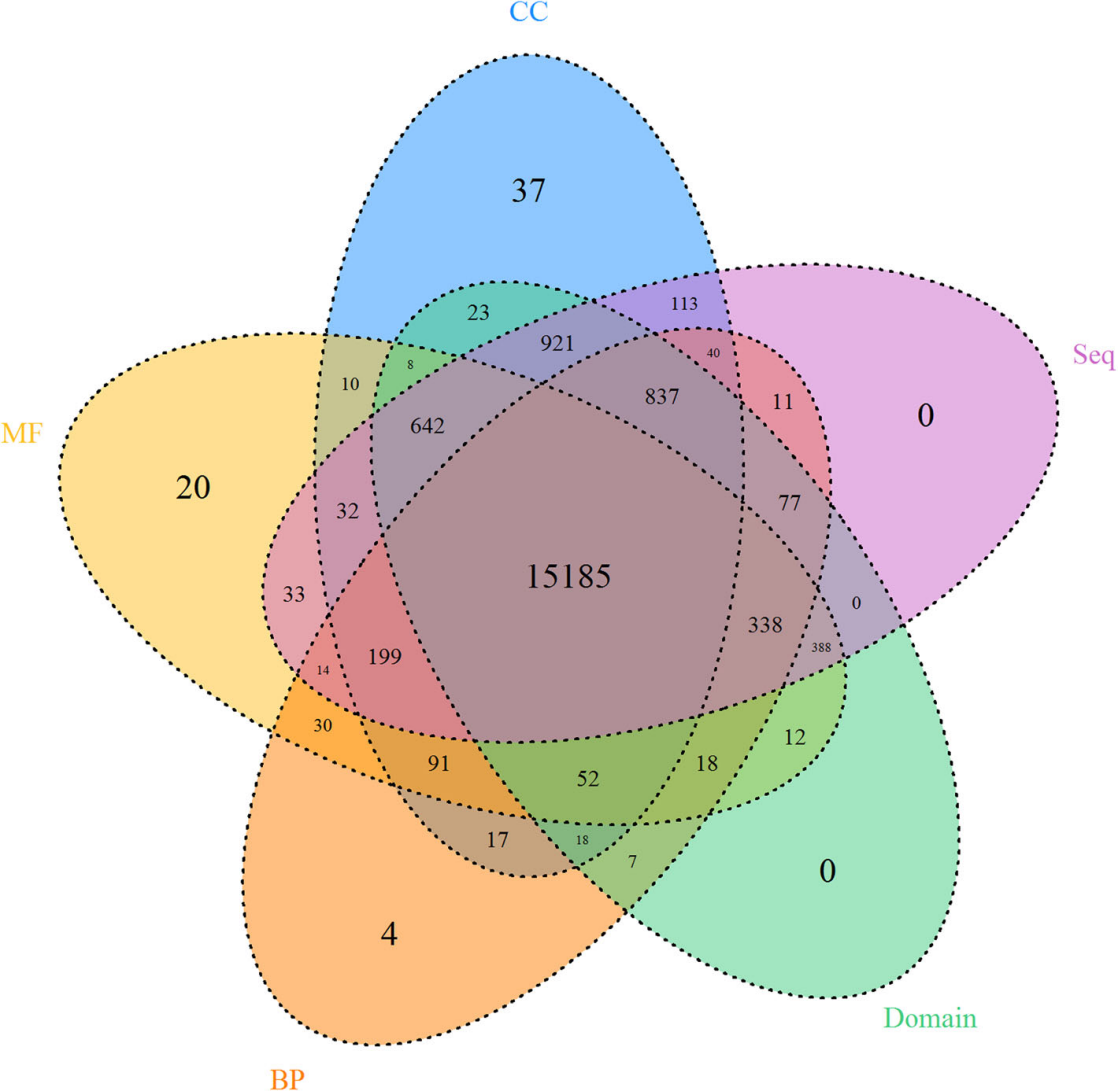
A brief statistic about the number of genes (proteins) annotated by the corresponding information

The overlap among the five aspects of annotation information about genes (proteins) above is unexpectedly large, as shown in Fig. 2. Numbers in the figure denote the number of genes that annotated by the corresponding information in each part, where CC, MF and BP denote corresponding annotations of genes. Seq and Domain denote amino acid sequences and domain of proteins.

### Integrating gene similarity networks based on SNF method

We have constructed five gene similarity networks based on BP, CC, MF, sequence and domain information of genes. In this subsection, we will employ SNF method [60] to integrate these five networks.

Suppose *W* ^(*m*)^ (here m = 1,2,3,4,5) denotes one of the adjacent matrices of gene similarity networks, we use Eq. (1) to compute the normalized weighted matrix of *W* ^(*m*)^, which can be defined as:

1 $$ {P}_{ij}^{(m)}=\left\{\begin{array}{l}\frac{W_{ij}^{(m)}}{2{\sum}_{k\ne i}{W}_{ik}^{(m)}}\kern0.5em if\ j\ne i\\ {}\kern2em \frac{1}{2}\kern1em if\ j=i\ \end{array}\right. $$

The normalization used here is free of the scale of self-similarity in the diagonal entries. It can avoid numerical instabilities and ∑_*j*_ *P*(*i*, *j*) = 1 still holds.

At the same time, we define the local kernel matrix ${S}_{i,j}^{(m)}$, which is calculated by Eq. (2)

2 $$ {S}_{ij}^{(m)}=\Big\{{\displaystyle \begin{array}{l}\frac{W_{ij}^{(m)}}{\sum_{k\in {V}_i^{(m)}}{W}_{ik}^{(m)}}\kern0.5em ifj\in {V}_i^{(m)},\\ {}\kern2em 0\kern2.5em \mathrm{otherwise}\end{array}}\operatorname{} $$

where ${V}_i^{(m)}$ denotes a set which contains *K* nearest neighbors of gene *i* in the matrix *W* ^(*m*)^. Since local similarities (high values) are more reliable than remote ones, we filter out the low similarity neighbors and set these similarities to zero. The *K* most similar genes for each gene in the networks are preserved. The local neighborhoods are further exploited to measure the local affinities among genes [61]. Therefore, *S* ^(*m*)^ keeps the local structure of *W* ^(*m*)^.

In summary, *P* ^(*m*)^ carries the full information about the similarity of each gene to all others, whereas *S* ^(*m*)^ only encodes the similarity to the *K* most similar genes. Here, *P* ^(*m*)^ and *S* ^(*m*)^ are called status matrices and kernel matrix [60], respectively.

To fuse the similarity networks, SNF takes the interactive process of the following update equation:

3 $$ {P}_{t+1}^{(m)}={S}^{(m)}\times \left(\frac{1}{M-1}\sum_{n\ne m}{P}_t^{(n)}\right)\times {\left({S}^{(m)}\right)}^T $$

where *m* is the index of corresponding adjacent matrices of similarity networks, and *t* is the iteration number. It should be noted that we perform normalization on ${P}_{t+1}^{(m)}$ as in Eq. (1) after each iteration. Another way to think of the updating rule (3) is

4 $$ {P}_{t+1}^{(m)}\left(i,j\right)=\sum_{h\in {V}_i^{(m)}}\sum_{l\in {V}_j^{(m)}}{S}_{i,h}^{(m)}\times {\left(\frac{1}{M-1}\sum_{n\ne m}{P}_t^{(n)}\right)}_{h,l}\times {S}_{j,l}^{(m)} $$

**Fig. 3:**
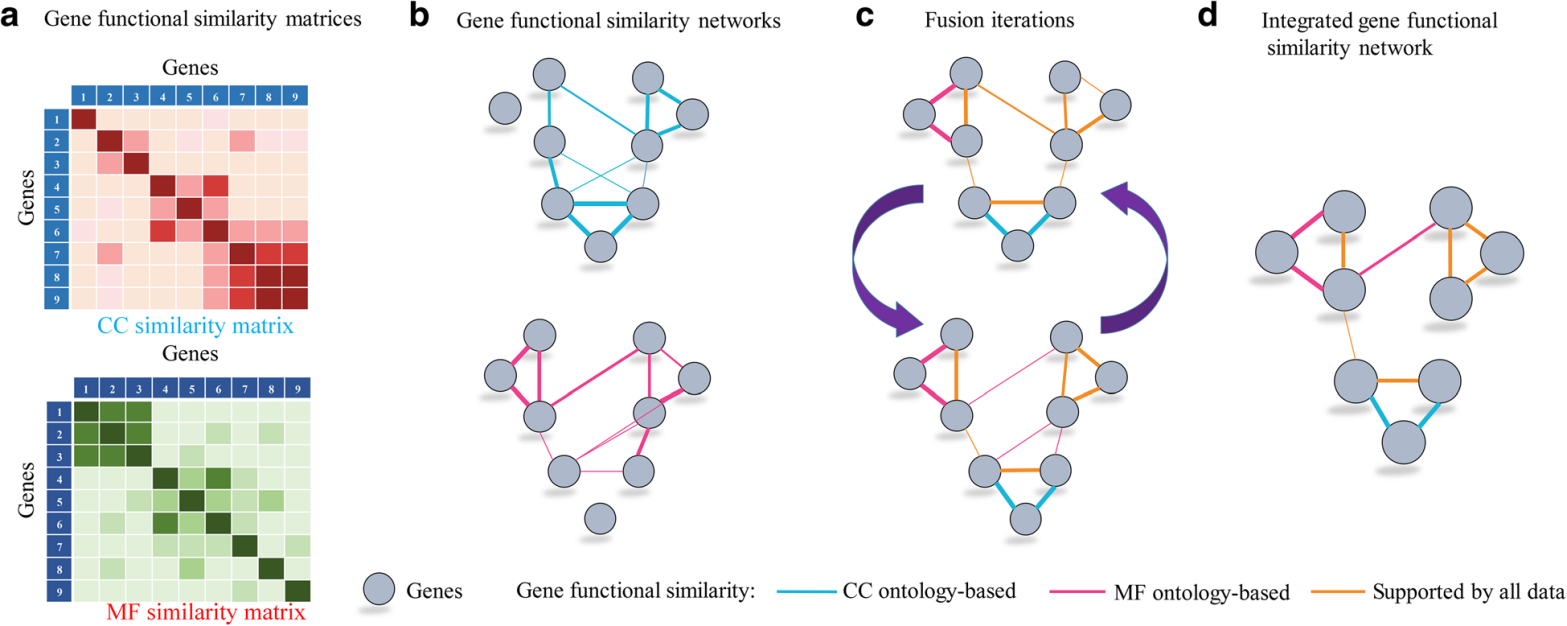
Illustrative example of SNF steps. (**a**) Gene-gene similarity matrices based on CC and MF ontology, respectively. (**b**) Gene functional similarity networks. Genes are represented by nodes and pairwise similarities between genes are represented by edges. (**c**) Network fusion by SNF updates iteratively, making them more similar with each step. (**d**) The iterative network fusion results in convergence to the final integrated network. Edge color indicates which data type has contributed to the given similarity

Because the similarity information is only propagated through the common neighborhood between genes, SNF is robust to noise existing in genome data. Besides, if two genes *g*_*i*_ and *g*_*j*_ have common neighbors in all of similarity matrices, it should be well believed that they have the high similarity. What’s more, SNF benefits the fact that even if *g*_*i*_ and *g*_*j*_ are not very similar in one data type, their similarity can be measured in another data type and this similarity information can be propagated through the fusion process [60, 62]. The illustrative example for fusing two networks based on SNF is shown in Fig. 3.

Finally, after *t* steps of iteration, these five matrices will converge to a single integrated matrix, which can be computed as:

5 $$ P=\frac{1}{M}\sum_{m=1}^M{P}_t^{(m)} $$

We obtain the **primary integrated gene similar network** in this step.

### Clustering coefficient-based threshold selection

The five gene similarity networks are fused as a primary integrated gene similar network, whose nodes represent the genes and edges represent the similarity between genes. However, there is still a serious problem needing to be addressed that how similar between two genes can be connected in the network. Because most molecular networks follow a power law or lognormal distribution [12], we should set an appropriate threshold to ensure that the primary integrated gene similarity network meets this demand. The similarity between genes which is greater than the proper threshold will be connected by edges. Otherwise, the similarity will be set to zero [46]. In this research, we adopt the clustering-coefficient-based threshold selection method to select a proper threshold for the primary integrated gene similarity network.

The clustering coefficient of a gene *i* in the network is defined as:

6 $$ {C}_i=\frac{2{E}_i}{k_i\left({k}_i-1\right)} $$

where *E*_*i*_ represents the number of edges between the *k*_*i*_ (>1) first neighbors of gene *i*. The clustering coefficient of a network is defined as the average clustering coefficient of its all nodes.

7 $$ C=\frac{1}{K}\sum_{k_i>1}{C}_i $$

where *K* denotes the total number of nodes in the network.

The threshold selection for a network can be regarded as a process, where edges are removed from the initially complete graph by gradually increasing the similarity threshold between genes. For each threshold *r*, we can construct a network by the means of filtering out the similarity lower than the threshold *r*. It is generally believe that the clustering coefficient of molecular networks, denoted by*C*(*r*), should be significantly higher than the that of the corresponding random network, which is denoted by *C*_0_(*r*).

Therefore, we formulate a discrete optimization problem, in which the cutoff threshold should meet the demand

8 $$ {C}^{\ast }=\underset{j}{\min}\left\{{r}_j:C\left({r}_j\right)-{C}_0\left({r}_j\right)>C\left({r}_{j+1}\right)-{C}_0\left({r}_{j+1}\right)\right\} $$

over a set of thresholds 0 = *r*_0_ < *r*_1_ <  ⋯  < *r*_*j* − 1_ < *r*_*J*_ = 1. In Eq. (8), *r*_*j* + 1_ = *r*_*j*_ + 0.001; *C*(*r*)and *C*_0_(*r*) denote the clustering coefficients of the gene similarity network and the corresponding random network at the threshold *r*, respectively. The aim of this procedure is to find the first local maximum, which means the first stop of monotonically increasing of *C*(*r*_*j*_) − *C*_0_(*r*_*j*_).

On the other hand, the clustering coefficient of a corresponding random network is determined by

9 $$ {C}_0=\frac{{\left({\overline{k}}^2-\overline{k}\right)}^2}{{\overline{k}}^3N} $$

where N is the total number of nodes in a network, $\overline{k}=1/N{\sum}_{i=1}^N{k}_i$ $\overline{k^2}=1/N{\sum}_{i=1}^N{k}_i^2$.

Finally, after threshold selection for the primary integrated gene similarity network, the **IGSN** that we need is constructed. It is represented as *G*(*V*, *E*, *t*), where *V* = { *g*_1_, *g*_2_,  ⋯ , *g*_N_} denotes the genes involving in IGSN, and *E* = {*e*_*ij*_ =  ≺ *g*_*i*_, *g*_*j*_ ≻ |*sim*(*g*_*i*_, *g*_*j*_) > *t*} represents the edges between genes with values greater than threshold *t*.

### Construction of the phenotype-gene bilayer network

**Fig. 4:**
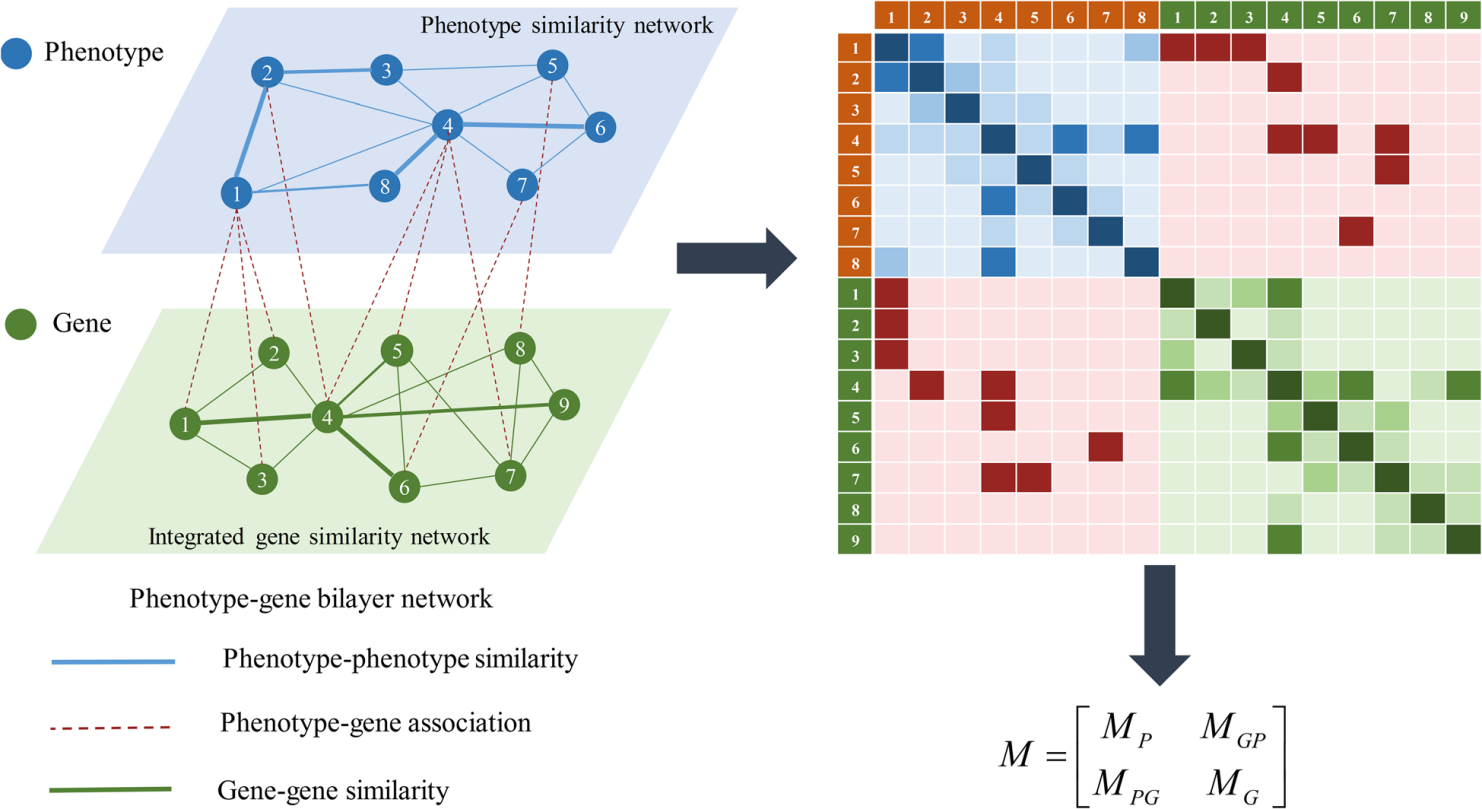
The construction process of phenotype-gene bilayer network

We have got three networks, which are phenotype similarity network, IGSN and phenotype-gene association network respectively. In this subsection, we make use of the three networks above to construct a phenotype-gene bilayer network. The construction process of phenotype-gene bilayer network is illustrated in Fig. 4.

Suppose *A*_*P*_(*m* × *m*), *B*_*GP*_(*m* × *n*) and *W*_*G*_(*n* × *n*) are adjacency matrices for phenotype similarity network, phenotype-gene association network and IGSN respectively, where *m* and *n* represent the number of phenotypes and genes in their respective networks. The adjacency matrix of the phenotype-gene bilayer network is denoted as

$$ A=\left[\begin{array}{cc}\hfill {A}_P\hfill & \hfill {B}_{GP}\hfill \\ {}\hfill {B}_{PG}\hfill & \hfill {W}_G\hfill \end{array}\right] $$ (10)

where *B*_*GP*_ is the transpose of *B*_*PG*_.

### Prioritizing candidate disease genes based on RWRB

The RWRB is a ranking algorithm, which simulates a random walker moving from the seed nodes to their immediate neighbors randomly and staying at the current node(s) based on the probability transition matrix [32, 63]. As for a random walk on the bilayer network, we first construct the transition matrix *M* based on matrix *A*, which is defined as

11 $$ M=\left[\begin{array}{cc}\hfill \lambda {M}_P\hfill & \hfill \left(1-\lambda \right){M}_{GP}\hfill \\ {}\hfill \left(1-\lambda \right){M}_{PG}\hfill & \hfill \lambda {M}_G\hfill \end{array}\right] $$

where *M*_*P*_, *M*_*GP*_ and *M*_*G*_ are the row-normalizing matrices of *A*_*P*_, *B*_*PG*_ and *W*_*G*_ respectively; *λ* controls the jumping probability between two similarity networks, which are phenotype similarity network and IGSN. Then the initial vector *P*(0) (at *t* = 0) can be defined as follows:

12 $$ P(0)=\left[\begin{array}{l}\left(1-\eta \right)u(0)\\ {}\kern1em \eta v(0)\end{array}\right] $$

where *u*(0) and *v*(0) denote the initial probability vector for phenotype similarity network and IGSN. The parameter *η* ∈ (0, 1) is used to weight the importance of phenotype similarity network and IGSN. The effect of the parameters *λ* and *η* on RWRB will be shown in the result section. *P*(*t*) represents a vector in which the *i*-th element holds the probability of finding the random walker on node *i* at step *t.*

Based on the vector *P*(0), *P*(*t*) and the transition matrix *M*, the probability vector at step *t* + 1 can be given by

13 $$ P\left(t+1\right)=\left(1-\gamma \right){M}^TP(t)+ rP(0) $$

where *γ* ∈ (0, 1) indicates the restart probability. At each step, the random walker has a probability *γ* to return the seed nodes.

After some steps, the walking process is converged if the change between *P*(*t*) and *P*(*t* + 1) is lower than 10^−6^. The steady probability *P*(∞) is represented as $$ P\left(\infty \right)=\left[\begin{array}{l}\left(1-\eta \right)u\left(\infty \right)\\ {}\kern1em \eta v\left(\infty \right)\end{array}\right] $$. As a result, genes which belong to the control set are ranked according to their probability scores in *P*(∞). Gene which has the maximum in *P*(∞) among all the control gene set is considered as the most probable gene that associates the phenotype.

### Evaluation metrics of prediction performance

Phenotypes in OMIM database mainly have three types [33, 35]: susceptible chromosomal locus and several related disease genes are known; susceptible locus is known, but no related genes are known; locus and related causal genes are unknown, but the phenotype is known. Therefore, we use three leave-one-out cross-validation experiments, i.e. *linkage interval*, *genome-wide scan* and ab initio, which are detailedly introduced and used in [35, 43], to validate our method.

Firstly, as for some phenotypes that susceptible chromosomal locus and several related disease genes are known, we take the cross validation against a *linkage interval* experiment [43]. In each round of validation, one phenotype-gene link is removed. We define the gene associates with the removed link as the held out gene. The phenotype and the rest disease genes related to this phenotype are used as the seed nodes. At the same time, we define the control gene set that consists of the held out disease gene and its 99 nearest genes according to the NCBI refGene location. The performance of RWRB is investigated by the capability to recover the held out disease gene from the control gene set. We call this as *linkage interval* experiment.

Secondly, since there are some phenotypes that have no susceptible chromosomal locus but have already experimental validated disease genes, we take the validation against genes in the genome-wide scale. In this experiment, we also remove a phenotype-gene relationship and use the rest disease gene associated with this phenotype as the seed nodes. Different to *linkage interval* experiment, the control gene set consists all the genes in the genome-wide scale except the held out disease gene. The performance of RWRB is investigated by the rank of held out gene in the control gene set. We call this as the *genome-wide scan* experiment.

Thirdly, as for some phenotypes without any known disease genes and susceptible chromosomal locus, we identify disease genes for these kinds of phenotypes from the whole-genome scale. In this experiment, we first remove all the associations between this phenotype and its disease genes, then run the RWRB algorithm which treats this phenotype as seed node. In this situation, the control gene set is defined as all the genes that in the whole networks. Similar to *genome-wide scan* experiment, the performance of RWRB is investigated by the rank of held out gene in the control gene set. We call this as ab initio experiment. The detail explanations for the three approaches have been described by Li [35] and Jiang [37].

At the same time, we also define three metrics to investigate the performance of RWRB. First is **number of successful predictions** (NSP). For each experiment above, in each round of validation, if the held out disease gene is ranked as top 1 among the control gene set, we consider it a successful prediction. Further, for a set of validation runs in each experiment, we sum up the **number of successful predictions** and treat it as a metric that represents effectiveness of algorithms. Second is the **mean rank ratio** (MRR), which is defined as the average rank ratios of all held genes in control gene sets in all validation runs. Third is the receiver operation characteristic (ROC) curve. We plots the sensitivity versus 1-specificity which subject to the threshold separating the prediction classes [10]. Sensitivity refers to the percentage of disease genes that are ranked above a particular threshold, while specificity refers to the fraction of control genes rank below the threshold. We vary the threshold from 0.0 to 1.0 with the scale 0.01, and draw the ROC curve. It is well accepted that smaller MRR and larger AUC and NSP values indicate better performance for a prioritization method [43].

## Results

First of all, we will investigate the performance of RWRB on three kinds of experiments. Then, we assess the effect of parameters in RWRB algorithm. After that, the proposed algorithm is compared with two similarity-based methods, which are CIPHER [33] and RWRH [35] and two feature-based methods which are PUDI [15] and PriDiGe [14]. Finally, we predict novel causal genes for Alzheimer’s disease and other common diseases based on RWRB algorithm.

### The performance of RWRB

**Fig. 5:**
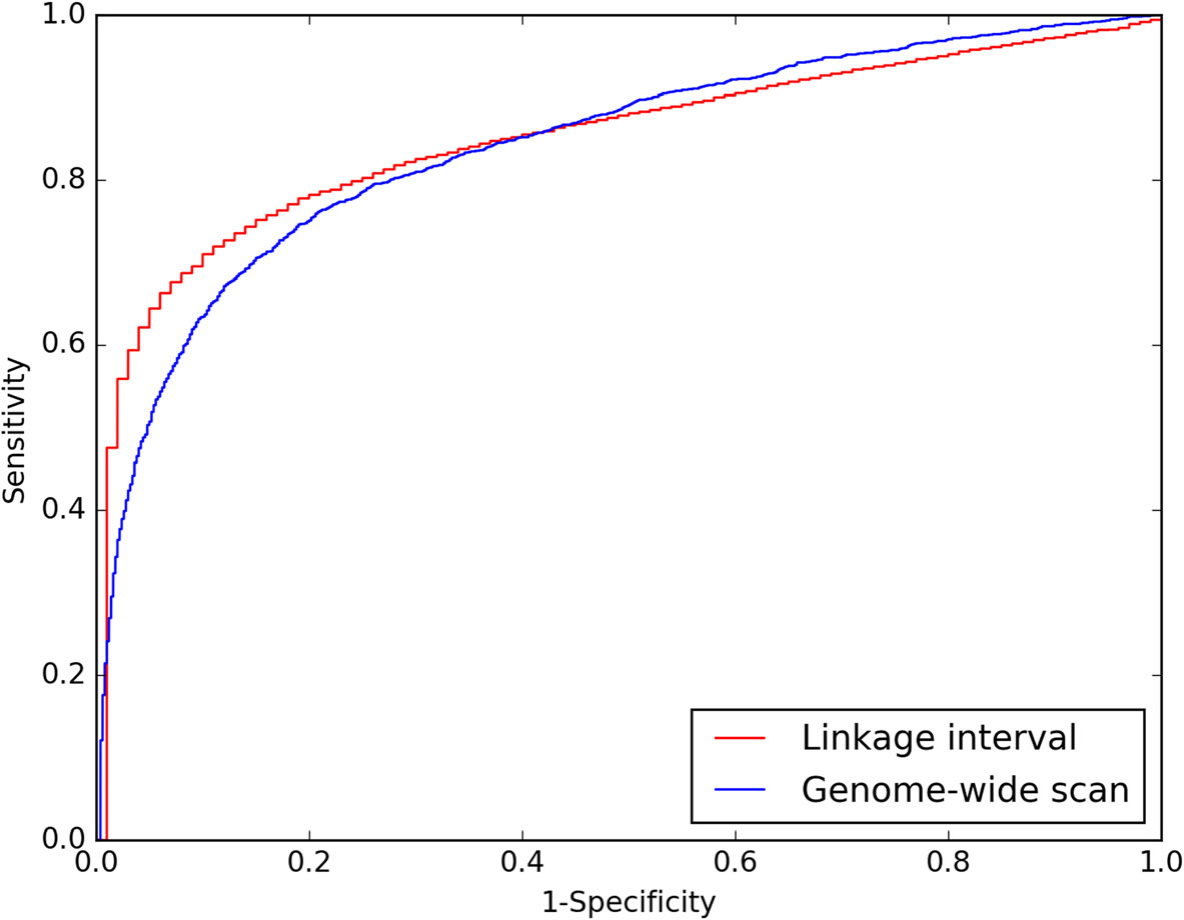
ROC curves of RWRB on *linkage interval* and *genome-wide scan* experiments

In this subsection, we will investigate the performance of RWRB on the three experiments using the three metrics. The detail results are shown in Table 1. The ROC curves on *linkage interval* and *genome-wide scan* experiments are shown in Fig. 5.

**Table 1:** The results of RWRB on the three experiments

Experiment	NSP	MRR	AUC
Linkage interval	1384	18.28	0.8505
Genome-wide scan	311	22.17	0.8417
ab initio	223	29.64	0.8144

As is shown in Table 1, the results of RWRB on NSP, MRR and AUC metrics for *linkage interval* experiment is 1384, 18.28, 0.8505, respectively. Then we further investigate the performance of RWRB on *genome-wide scan* experiment and obtain a NSP of 311, a MRR of 22.17 and an AUC of 0.8417. In the end, we perform the cross-validation approach against ab initio experiment. The results on NSP, MRR and AUC are 223, 29.64 and 0.8144, respectively.

As is known to us, a random guess will yield a MRR of 50%, and an AUC of 50%, suggesting that the effectiveness of RWRB in uncovering disease gene. Meanwhile, the results also show the reliability of IGSN.

**Fig. 6:**
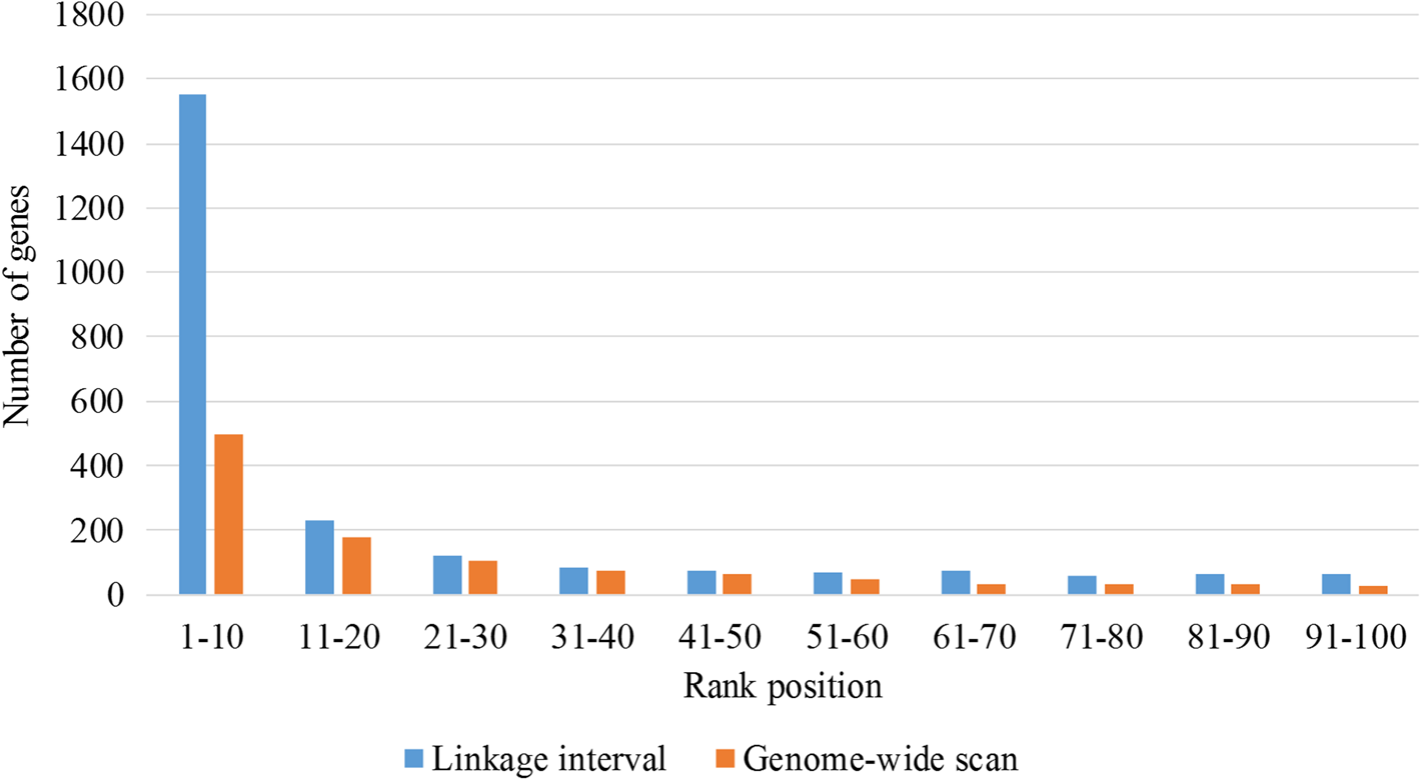
The distribution of disease genes ranked in top 100

Then we further analyze the detail distribution of disease genes ranked in the control gene set for *linkage interval* and *genome-wide scan* experiment. The results are presented in Fig. 6. As for *linkage interval* experiment, we find that there are 1554 disease genes ranking in top 10, where 1384 disease genes are rank one. 230 disease genes are ranked between 11 and 20, and 157 disease genes are ranked between 30 and 50.

As for *genome-wide scan* experiment, there are 498 disease genes ranking between 1 and 10. The number of disease genes between 11 and 50 is 422. As we can see from the results, most of held out genes can be rank in top 100. The results on three experiments demonstrate that RWRB has a high accuracy in inferring disease genes on the genomic scale.

### Effect of parameters on RWRB

There are totally three parameters in RWRB, which are *γ λ* and *η*. The parameter *γ* denotes the restart probability in Eq. (1). It has been well accepted that the parameter *γ* has a slight effect on the results and here we fix it at 0.7 [35]. Next, we will investigate the influence of parameter *λ* and *η* for RWRB on the NSP metric.

The parameter *λ* represents the jumping probability between phenotype similarity network and IGSN. According to [35], larger λ will introduce more mutual information between phenotype similarity network and IGSN. To investigate the effect of this parameter on the performance of RWRB, we tested our algorithm on different values of λ ranging from 0.1 to 0.9 with an increment of 0.1.

Results are shown in Table 2. The performance is improved with the increase from 0.1 to 0.6 on the whole. However, the performance is slightly decreased from 0.6 to 0.9. As for the *linkage interval* experiment, RWRB gets the best performance at λ = 0.6, while RWRB gets the largest NSP at λ = 0.7 on the *genome-wide scan* experiment. The best results for ab initio experiment is 225 when λ = 0.6. Therefore, we suggest that the best λ value is 0.6 or 0.7 for RWRB on the experiments above. Results demonstrate that the RWRB algorithm successfully makes the best use of the relationships between phenotype similarity network and IGSN.

**Table 2:** Performances of RWRB at different values of λ on NSP metric

λ	Linkage interval	Genome-wide scan	ab initio
0.1	1306	295	165
0.2	1320	299	169
0.3	1337	304	181
0.4	1349	309	209
0.5	1384	311	223
0.6	**1393**	317	**225**
0.7	1386	**319**	211
0.8	1361	308	206
0.9	1357	304	174

To validate the effect of parameter λ on RWRB at different values, we fix η at 0.5. Best results are in bold

As is known to us, η controls the impact of seed phenotypes and seed genes in the initial vector. To validate the effect of parameter η on RWRB, we tested our algorithm on different values of η ranging from 0.1 to 0.9 with the scale 0.1. We run RWRB on *linkage interval* and *genome-wide scan*, ab initio experiments, and evaluate its performance on the NSP metric. As is shown in Table 3, the performance is improved with the increase from 0.1 to 0.6 on both experiments. However, the performance is slightly decreased from 0.6 to 0.9. As a result, the algorithm performs best when η at 0.6. This suggests that IGSN is more importance than phenotype similarity network for RWRB.

**Table 3:** Performances of RWRB at different values of η on NSP metric

η	Linkage interval	Genome-wide scan	ab initio
0.1	1286	299	175
0.2	1310	307	187
0.3	1344	310	193
0.4	1368	310	206
0.5	1384	311	223
0.6	**1392**	**319**	**227**
0.7	1391	317	217
0.8	1378	315	203
0.9	1354	306	172

To we validate the effect of parameter η at different values, we fix λ at 0.5. Best results are in bold

### Comparison with similarity-based methods

We compare RWRB with similarity-based methods which are RWRH [35] and CIPHER [33], respectively. The author [33] defines two topological distance on the basis of two different neighborhood systems: shortest path (SP) and direct neighbor (DN). Therefore, two versions of CIPHER are represented as CIPHER-SP and CIPHER-DN, respectively. The results of each method on NSP metric are presented in Table 4.

**Table 4:** The performance of each method on the NSP metric

Algorithms	Linkage interval	Genome-wide scan	ab initio
RWRH	814	245	201
CIPHER-SP	709	153	140
CIPHER-DN	765	165	157
RWRB	**1384**	**311**	**223**

Note: Best results are in bold

Because the number of phenotype-gene associations in RWRB, CIPHER and RWRH models are different, we compute the successful prediction percentages for each method, which is defined as the ratio between NSP and the total phenotype-gene associations in their corresponding datasets. The experimental results are listed in Table 5.

**Table 5:** The successful prediction percentages for each method

Algorithms	Linkage interval	Genome-wide scan	ab initio
RWRH	0.56	**0.17**	**0.14**
CIPHER-SP	0.49	0.11	0.10
CIPHER-DN	0.52	0.12	0.11
RWRB	**0.58**	0.13	0.09

Note: Best results are in bold

As for the *linkage interval* experiment, RWRB gets 1384 successful predictions, while RWRH, CIPHER-SP and CIPHER-DN obtain 814, 709, 765 successful predictions, respectively. The percentage of successful prediction for RWRB is 0.58 which is the highest in all three methods.

As for the *genome-wide scan* experiment, the control gene set is defined as the whole genes in IGSN. RWRB get 311 successful predictions, while RWRH, CIPHER-SP and CIPHER-DN obtain 245, 153, 165 suc cessful predictions, respectively. Then number of successful predictions of RWRB is largest in the three methods. However, the percentage of successful predictions for RWRB is 0.13 which is lower than that of RWRH (0.17).

On the ab initio experiment, there are 223 successful predictions by RWRB, while RWRH, CIPHER-SP and CIPHER-DN successfully predicted 201,140 and 157 cases, respectively. However, the percentage of successful predictions of RWRH is the highest in the three methods which is 0.14, whereas the other three methods are almost neck and neck.

### Comparison with feature-based methods

At the same time, we compare RWRB with two feature based methods which are PUDI [15] and PriDiGe [14]. Here we only compare the precision (p), recall (r) and F-measure (F) of these three methods, since they are from different type of methods.

The metrics about precision, recall and F-measure for PUDI and ProDiGe have been introduced by Yang [15]. Here, we will also use these metrics to evaluate the performance of RWRB on *linkage interval* experiment. In the experiment, we take the leave-one-out cross-validation method. For the precision of RWRB, we define it as the ratio between NSP and the number of all validation runs. For the recall of RWRB, we define it as the ratio between the number of the held out genes whose rank proportions are higher than 0.5 and the number of all the held out genes. The F-measure is the harmonic mean of precision and recall, which is defined as *F* = 2^∗^ *p* ^∗^r/(*p* + *r*).

The results for PUDI, ProDiGe and RWRB are shown in Table 6. From the results, we can find that RWRB achieves 82.3% recall and ranks first in the three methods. Method PUDI wins 72.3% precision which is 13.7 and 0.9 better than RWRB and ProDiGe method, respectively. At the same time, method PUDI achieves 76.5% F-measure which is 2.0% and 10.2% better than RWRB and ProDiGe method, respectively. In this group experiment, method PUDI performs best and RWRB ranks second overall.

**Table 6:** Overall comparison among different methods

Methods	Precision	Recall	F-measure
PUDI	72.3	81.0	**76.5**
ProDiGe	**72.4**	75.9	74.1
RWRB	58.6	**82.3**	69.4

Note: Best results are in bold

### Prioritizing Alzheimer’s disease and other common disease genes by RWRB: A case study

In this subsection, we will use RWRB to predict novel causal genes of interested diseases. To validate the effectiveness of our method, we will check whether our predicted disease genes have been already found to associate with the diseases in literature. Here, we select 16 multifactorial diseases which are used in [37] and list the top 10 candidate genes for each disease. The results are shown in Table 7. Here, we only select Alzheimer’s disease (AD) as the case study to verify the performance of RWRB.

**Table 7:** Top-10 predicted causal genes of 16 multifactorial diseases

Phenotype name	Phenotype ID	Top ten predictions for each phenotype by RWRB									
Alzheimer’s disease	104,300	**NOS2**	**NOS1**	APBB3	**APBB1**	EPX	LPO	APLP1	**PGBD1**	POR	MTRR
Breast cancer	114,480	RB1	PTEN	AR	TP63	TP73	SDHD	BUB1B	GNAS	PHB2	TSC1
Colon cancer	114,500	RB1	PTEN	SDHD	BRCA1	MLH1	MSH2	BRCA2	CREBBP	TP63	TP73
Diabetes mellitus	125,853	INSR	APOA5	VDR	HMGA2	SLC2A2	LPL	GHR	INS	USF1	LMNA
Gastric cancer	137,215	IL36A	IL36G	IL1A	IL1F10	IL37	IL36B	IL36RN	APC	IL18	MSH2
Atrial fibrillation	147,050	WAS	SELL	PAFAH2	SELE	TIMD4	HAVCR2	IL13	IKBKG	TNFRSF13B	ICOS
Prostate cancer	176,807	HIP1R	BRCA1	TP53	STK11	FGFR3	ZFHX4	SDHD	RNASEL	PRODH	MSH2
Schizophrenia	181,500	SYN3	SYN1	MAPT	DDO	PRNP	CHI3L2	APOL3	CHIA	CHIT1	APOL1
Leukemia	190,685	FLNA	FGFR2	RET	GLI3	NF1	COL1A1	COL2A1	EVC	TBX1	FLNB
Lung cancer	211,980	TP53	CDKN2A	RB1	SDHD	NRAS	CYP2D6	BRCA1	CYLD	DICER1	PTEN
Zellweger	214,100	FGFR2	FLNA	COL2A1	MECP2	FGFR3	FLNB	TP63	GLI3	GJA1	COL11A1
Leukemia	253,310	SMN1	GBA	LMNA	VAPB	ATP7A	ALS2	COL6A2	BSCL2	DCTN1	COL2A1
Asthma	600,807	IL2RG	SCGB1D2	SCGB1D4	SCGB1D1	PAFAH2	SBDS	WAS	IGHM	HPS1	ALOXE3
Leukemia	601,626	BCR	PDGFRB	PRF1	KMT2A	BRCA2	MPL	MLLT1	MCL1	MLLT6	RPS14
Obesity	601,665	FFAR4	GNAS	SLC6A14	ASIP	ENPP3	SDC1	ENPP2	SDC2	SDC4	MLN
Tuberculosis	607,948	CD2AP	C5	SCNN1B	CFTR	TICAM2	FAM218A	TLR1	TLR4	TLR6	SOCS2

Note: Predicted disease g﻿enes which are supported by literature are in bold﻿ for Alzheimer’s disease

AD is a progressive disease that usually starts slowly and gets worse over time. In general, it causes 60% to 70% of cases of dementia. The cause of AD has not been completely understood so far. The primary task is to discover the disease genes to understand the nosogenesis of genetic disease. There are many phenotypes for AD. Here we select 104,300 as target phenotype to prioritize disease gene. The corresponding susceptible region for MIM:104,300 is 6p22.

As is shown in Table 7, the first prediction of RWRB for MIM:104,300 is NOS2, which plays an important role in neuroinflammation by generating nitric oxide (NO), a critical signaling and redox factor in the brain [64]. Further, the levels of NO fall in the brain to a threshold may promote Aβ mediated damage. The predicted gene NOS2 has a large impact on AD. The second prediction gene for MIM:104,300 is NOS1. In the brain and peripheral nervous system, nitric oxide displays many properties for a neurotransmitter. The author [65] suggests that short alleles of the NOS1 exon 1f–VNTR interacting with the epsilon 4 allele tend to markedly increase the AD risk [65]. The fourth predicted gene for AD is APBB1. A trinucleotide deletion of the *APBB1* gene was a factor protecting against late-onset AD. Cousin [66] reported the results of a case/control study and confirmed this relationship. The eighth prediction is gene *PGBD1*. It locates at 6p22 which is the suspectable region of MIM:104,300. What’s more, it currently shows significant association in AlzGene according to Genome-wide association study. Its gene product is specifically expressed in the brain and has been identified as the key factors of AD. The results above show that the combination of the similarity network integration and the identification algorithm can successfully predict candidate genes for interested disease.

## Conclusions and discussion

In this paper, we propose a novel method, named RWRB, to infer causal genes of interested diseases. We firstly construct five gene similarity networks based on five different types of genome data. Then we employ SNF method to integrate these gene similarity networks and get IGSN. After that, we perform RWRB to prioritize disease genes. RWRB is compared with the state-of-the-art models and achieves a better performance on most evaluation metrics. Next, we will discuss the highlights of this article.

### The advantages of IGSN

The main object of our research is to overcome two drawbacks of current PPI networks, i.e., their low reliability and coverage. As a result, we construct the IGSN in this research. Firstly, since IGSN is fused based on the five gene (protein) similarity networks, its reliability should be higher than existing that of PPI networks. The prioritization of disease genes can be benefited from IGSN. Secondly, IGSN can significantly improve the coverage of human genes comparing current PPI networks. It covers 19,065 genes, which is twice the number of genes in HPRD network. Therefore, the number of phenotype-gene associations in RWRB algorithm is 2386, which is almost twice that in RWRH and CIPHER methods whose number is 1444. As a result, the proposed method can make the best use of phenotype-gene associations in OMIM database. Thirdly, since IGSN is a single network which integrates multiple gene similarity networks, there is no need for it to assign weight values to different subnetworks.

### Threshold selection for IGSN

**Fig. 7:**
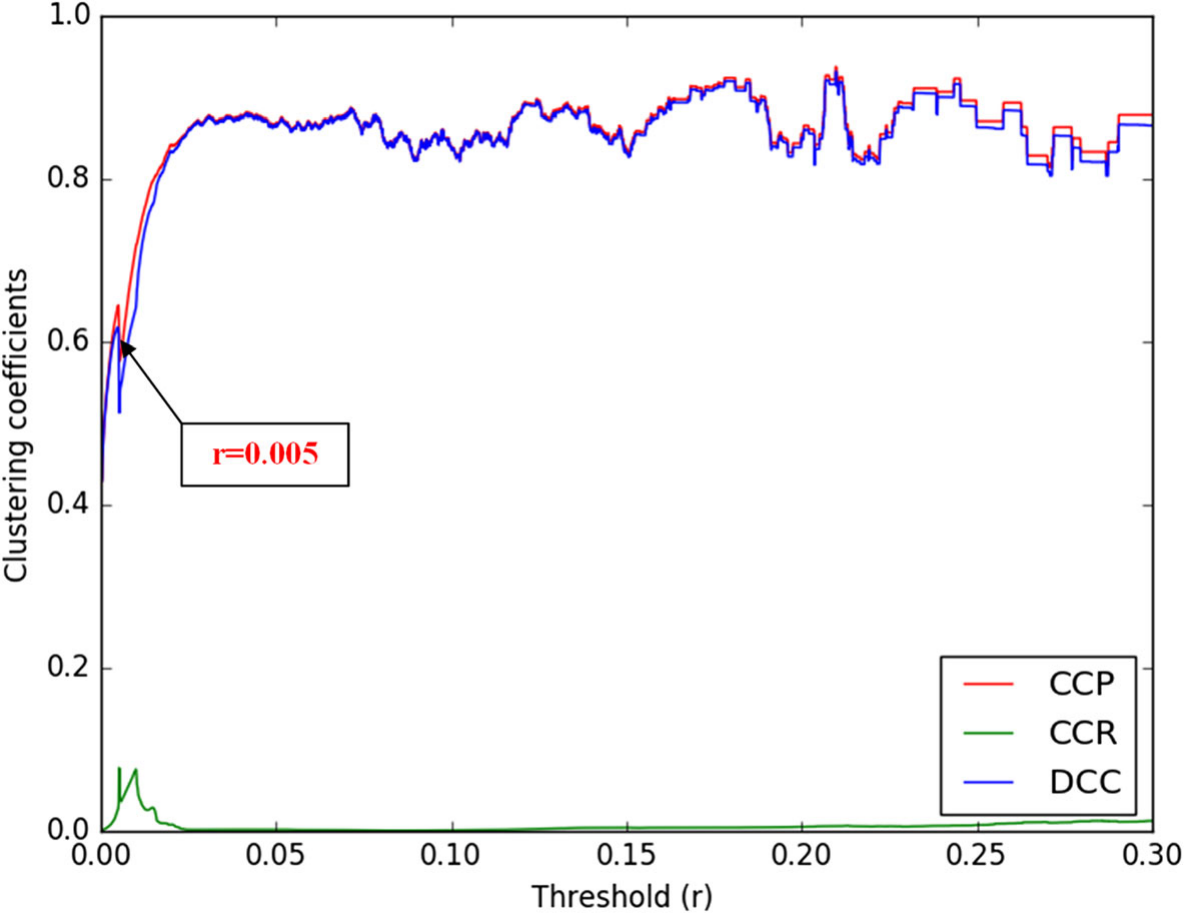
Cluster coefficient under each threshold for primary integrated gene similarity network. Black arrow points to the first peaks of the curve and rectangular boxes show the corresponding threshold value. Red curve represents the cluster coefficient of the primary integrated gene similarity network (CCP), and green curve denotes the cluster coefficient of the corresponding random network (CCR) at different thresholds. Blue curve depicts the difference of cluster coefficient (DCC) between the two networks above. In this experiment, we select the best threshold at *r* = 0.005, and construct IGSN based under this threshold

The threshold selection is very important to the quality of IGSN. This is because the threshold affects reliability of IGSN, and may further determine the performance of RWRB. As shown in Fig. 7, the first stop of monotonically increasing of DCC (See legend of Fig. 7) occurs at *r* = 0.005, which indicates that this threshold is the most appropriate value to construct IGSN. Under this threshold, the IGSN has 19,065 genes in our experiment.

We further investigate the degree distributions of IGSN under the selected threshold. Many previous studies [67] have found that distribution of node connectivity of molecular networks follows a power law. However, some other research [68] argued that there are some distributions, such as the lognormal distribution, which can also depict the degree distribution better than power law. In this research, we employ two models, which are Gaussian distribution and Lognormal distribution, to investigate the distributions of IGSN. In order to increase contrast, we import two other leading PPI networks, which are BioGRID and HPRD networks.

**Fig. 8:**
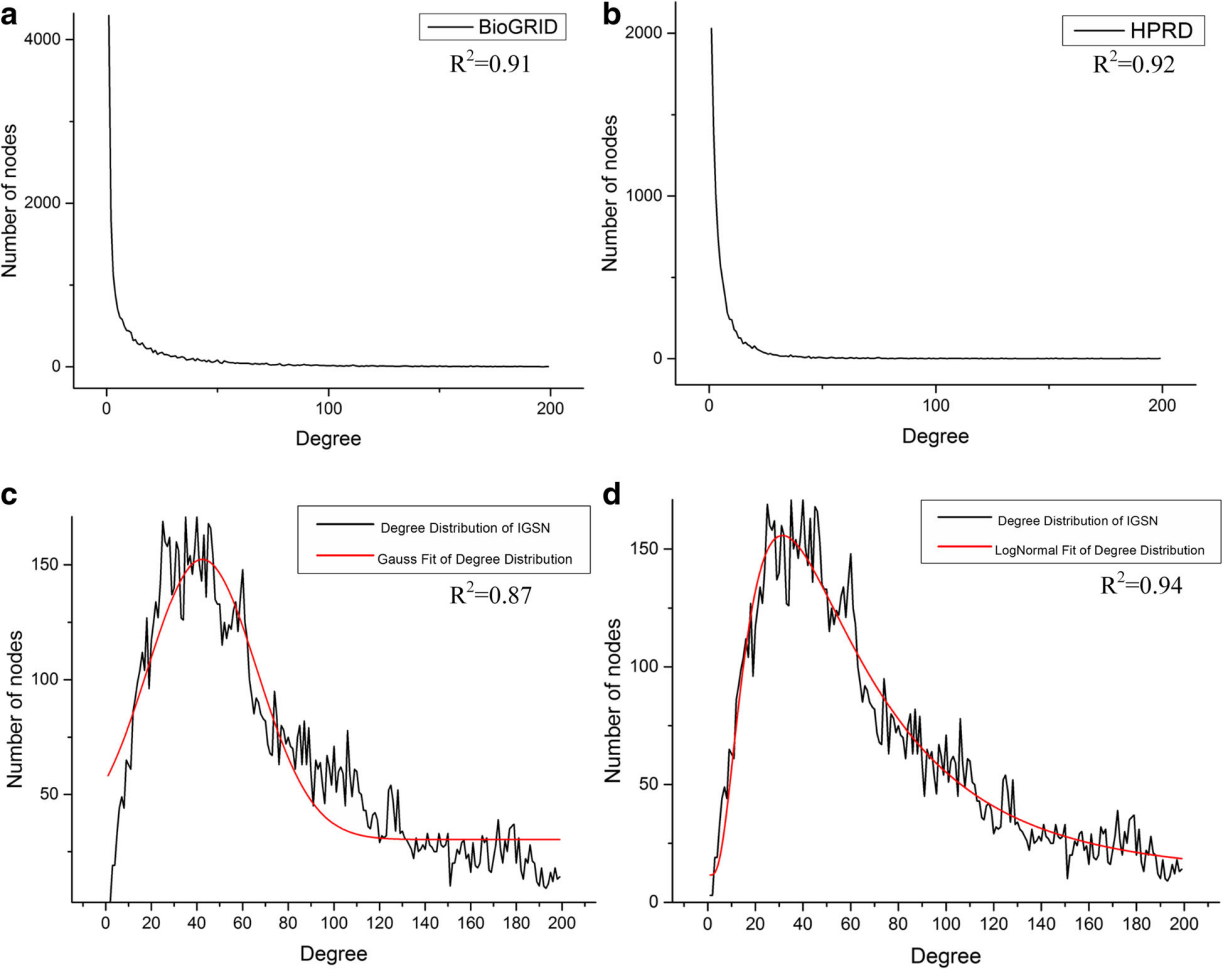
The graphic view of degree distribution fitting results for BioGRID (**a**), HPRD (**b**) and IGSN (**c**, **d**). According to their performance on R^2^, the results for IGSN fitting the Gaussian and Lognormal distribution are 0.87 and 0.92, shown with (**c**) and (**d**) respectively, while the results for BioGRID (**c**) and HPRD (**d**) are 0.91 and 0.92 respectively

The fitting performance on the distributions for each network is represented by R-squares (R^2^). R^2^ provides a measure of how well the data fits a certain model. As is shown in Fig. 8, we find that the degree of IGSN fits the lognormal distribution best, while BioGRID and HPRD prefer to fit the power law distribution. As is shown in Fig. 8 (c) and (d), the R^2^ results of IGSN for Gaussian and Lognormal distribution are 0.87 and 0.94, respectively. The R^2^ results of BioGRID and HPRD for fitting Power law are 0.91 and 0.92, respectively, which are shown in Fig. 8 (a) and (b). The degree distribution result shows that IGSN has the characteristics of molecular networks, rather than those of random networks. Therefore, IGSN is a meaningful biological network.

In the future, our research should further be improved from the following aspects. First, other genomic data of genes needs to be integrated. Although we have measured the similarity between genes based on five types of genome data, other information of genes is needed to be integrated to the similarity networks. Second, how to fuse the different similarity networks properly is important to the ultimate integrated network. Many previous studies have attempted to integrate different semantic similarity network and gene expression networks. However, some methods only assign equal weighted to these networks and simply add them together, while some others apply these networks separately. The SNF method used in this article may overcome the drawbacks above. However, the identification of the integrated network is not a trivial assessment because there is no direct way to ascertain its rationality and correctness. In our research, we resort to degree distribution of integrated network and find it fit the lognormal distribution best. This only shows the rationality from one property of the integrated network. Therefore, we need to study more fused methods of network further and make the integrated network be in line with the characteristics of biological networks.
